# Supplementary material for: A CircRNA–miRNA–mRNA Network for Exploring Doxorubicin- and Myocet-Induced Cardiotoxicity in a Translational Porcine Model
Source: Biomolecules. 2023 Nov 27;13(12):1711. doi: 10.3390/biom13121711 (PMC10741657; doi:10.3390/biom13121711)
Supplement: Supplementary file 1 [file biomolecules-13-01711-s001.zip › Supplementary Figure S1.pdf]

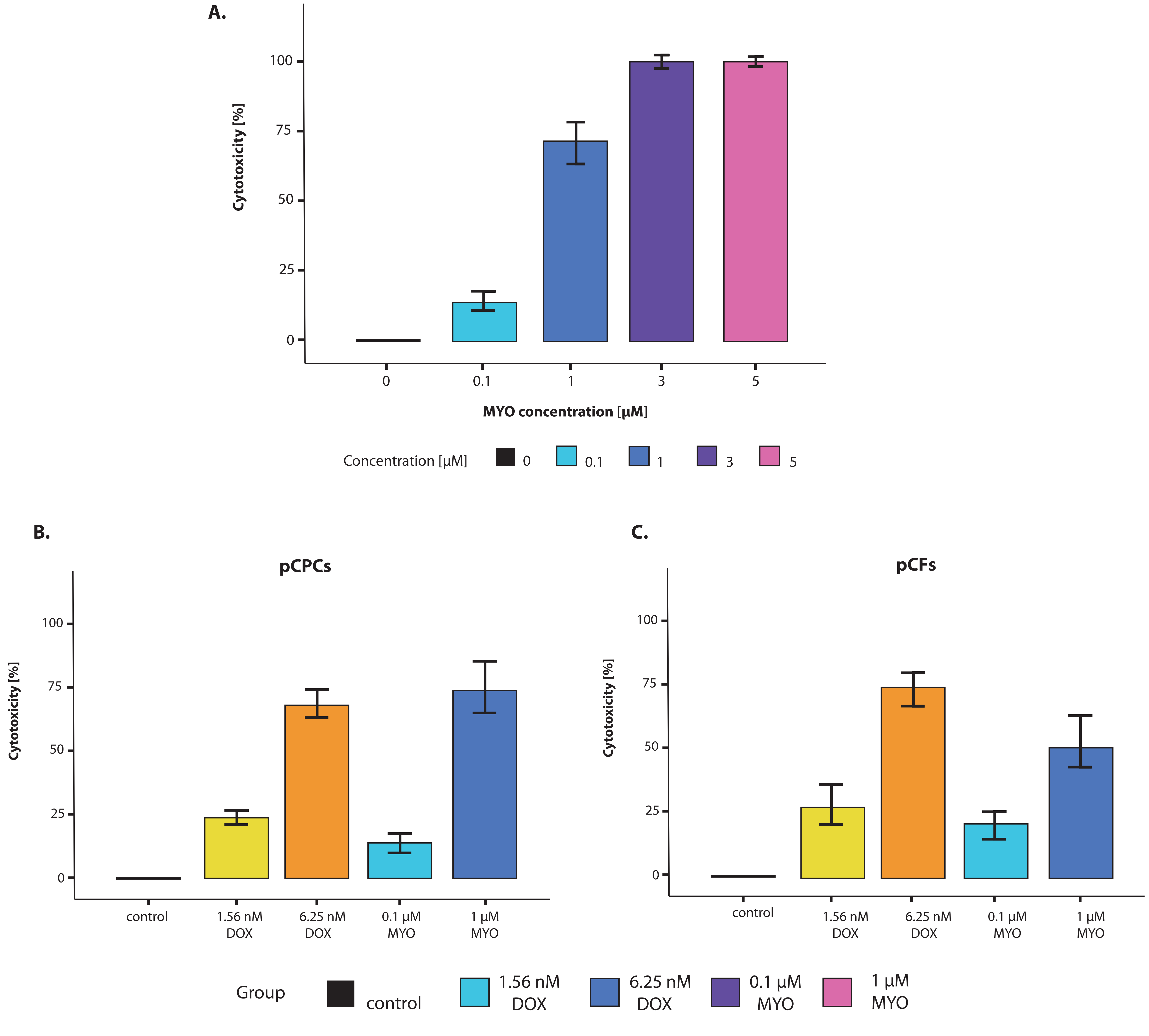

Supplementary Figure S1 Concentration finding of Doxorubicin and Myocet in pCPCs and pCFs *in vitro* via cytotoxicity assay
A. Cytotoxicity (in %) in pCPCs with increasing concentrations of MYO
B. Cytotoxicity (in %) in pCPCs with different DOX and MYO concentrations compared to untreated cells (control)
C. Cytotoxicity (in %) in pCFs with different DOX and MYO concentrations compared to untreated cells (control)
n = 6 (technical replicates) per group
