# Supplementary material for: A CircRNA–miRNA–mRNA Network for Exploring Doxorubicin- and Myocet-Induced Cardiotoxicity in a Translational Porcine Model
Source: Biomolecules. 2023 Nov 27;13(12):1711. doi: 10.3390/biom13121711 (PMC10741657; doi:10.3390/biom13121711)
Supplement: Supplementary file 1 [file biomolecules-13-01711-s001.zip › Supplementary Figure S2.pdf]

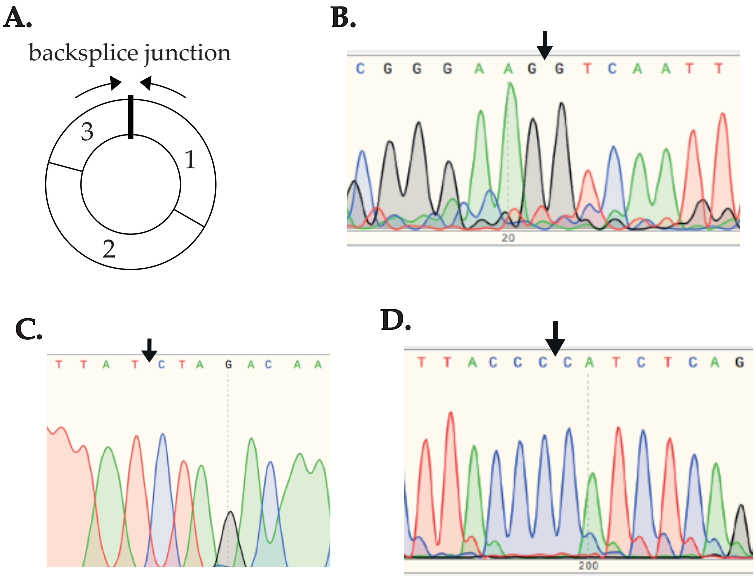

Supplementary Figure S2. The presence of backsplice junctions (BSJ) of circRNA in cell culture experiments confirmed using Sanger sequencing.

A. CircRNA formation. 1,2 and 3 represent exons, the thick black line represents the backsplice junction, the arrows represent the direction of forward and reverse primers needed for the correct amplification of the BSJ

B. Confirmed presence of BSJ in circ-MT:3033|3289, the arrow indicates the location of the BSJ

C. Confirmed presence of BSJ in circ-MT:3070|3478, the arrow indicates the location of the BSJ

D. Confirmed presence of BSJ in circ-7:22870230|22976632, the arrow indicates the location of the BSJ
