# Supplementary figures and images for: A CircRNA–miRNA–mRNA Network for Exploring Doxorubicin- and Myocet-Induced Cardiotoxicity in a Translational Porcine Model
Source: Biomolecules. 2023 Nov 27;13(12):1711. doi: 10.3390/biom13121711 (PMC10741657; doi:10.3390/biom13121711)

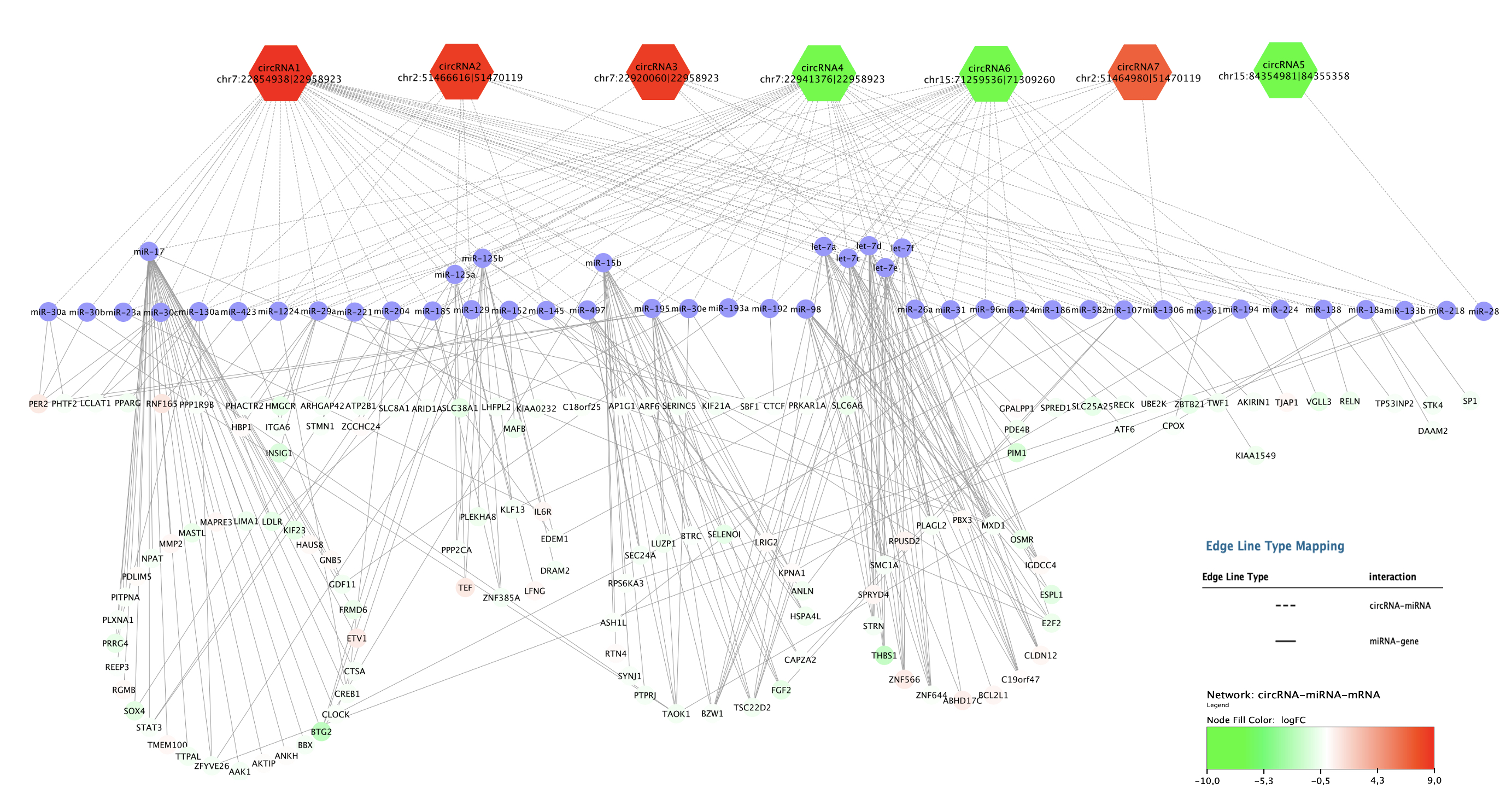

Supplement: Supplementary file 1 [file biomolecules-13-01711-s001.zip › Supplementary Figure S3.jpg]

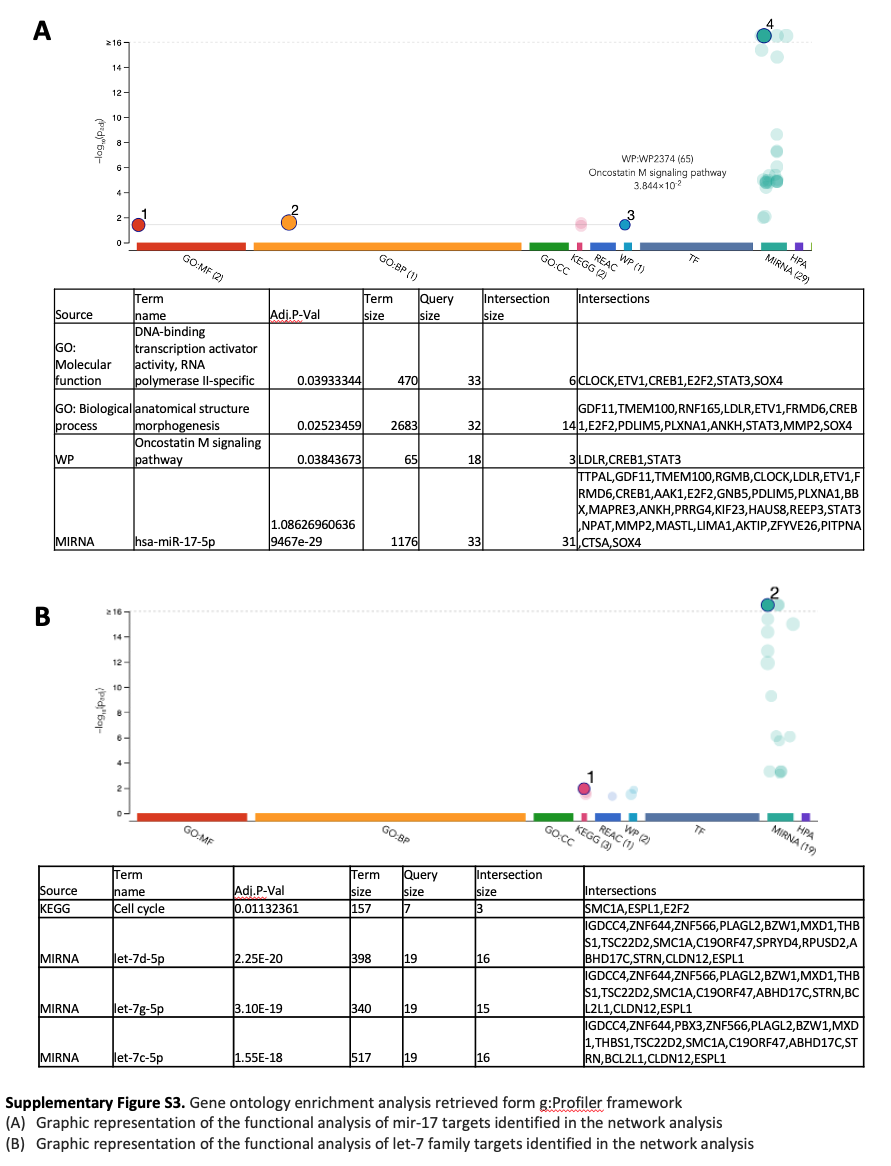

Supplement: Supplementary file 1 [file biomolecules-13-01711-s001.zip › Supplementary Figure S4.png]
